# Neopterin and Soluble CD14 Levels as Indicators of Immune Activation in Cases with Indeterminate Pattern and True Positive HIV-1 Infection

**DOI:** 10.1371/journal.pone.0152258

**Published:** 2016-03-31

**Authors:** Hayriye Kırkoyun Uysal, Pari Sohrabi, Zafer Habip, Suat Saribas, Emre Kocazeybek, Fatih Seyhan, Reyhan Calışkan, Esad Bonabi, Pelin Yuksel, Ilhan Birinci, Omer Uysal, Bekir Kocazeybek

**Affiliations:** 1 Department of Medical Microbiology, Istanbul Medical Faculty, Istanbul University, Istanbul, Turkey; 2 Istanbul Public Health Laboratory, Istanbul, Turkey; 3 Department of Medical Microbiology, Cerrahpasa Medical Faculty, Istanbul University, Istanbul, Turkey; 4 Medical Faculty of Marmara University, Istanbul, Turkey; 5 Istanbul Leprosy Dermatology and Venereology Hospital, Istanbul, Turkey; 6 Istanbul Aydın University - Health Services Vocational School Of Higher Education, Istanbul, Turkey; 7 Turkish Red Crescent, Istanbul, Turkey; 8 Deparment of Biostatistics, Medical School of Bezmialem Vakif University, Istanbul, Turkey; University of Malaya, MALAYSIA

## Abstract

**Background:**

We aimed to evaluate the roles of the plasma immune activation biomarkers neopterin and soluble CD14 (sCD14) in the indirect assessment of the immune activation status of patients with the indeterminate HIV-1 (IHIV-1) pattern and a true HIV-1-positive infection (PCG).

**Methods:**

This cross-sectional and descriptive study included eighty-eight patients with the IHIV-1 pattern, 100 patients in the PCG, and 100 people in a healthy control group (HCG). Neopterin and sCD14 levels were determined by competitive and sandwich ELISA methods, respectively.

**Results:**

Mean neopterin and sCD14 levels among those with the IHIV-1 pattern were significantly lower than among the PCG (*p* < 0.001 and *p* = 0.001, respectively), but they were similiar to those in the HCG (*p* = 0.57 and *p* = 0.66, respectively. Mean neopterin and sCD14 levels among the PCG were found to be significantly higher than among those with the IHIV-1 pattern (*p* < 0.001 and *p* = 0.001, respectively) and among those in the HCG (*p* = 0.001, *p* < 0.001, respectively). Neopterin did not have adequate predictive value for identifying those in the PCG (area under the curve [AUC] = 0.534; 95% CI, 0.463–0.605; *p* = 0.4256); sCD14 also had poor predictive value but high specificity (100%) for identifying those in the PCG (AUC = 0.627; 95% CI, 0.556–0.694; *p* = 0.0036).

**Conclusions:**

While low levels of these two biomarkers were detected among those with the IHIV-1 pattern, they were found in high levels among those in the PCG. These two markers obviously cannot be used as a sceening test because they have low sensitivies. Taken together, we suggest that neopterin and sCD14 may be helpful because they both have high specificity (92%-100%) as indirect non-specific markers for predicting the immune activation status of individuals, whether or not they have true positive HIV-1.

## Introduction

Neopterin is a purine nucleotide which forms as results of the guanosine triphosphate (GTP) catabolism. Levels increase in pathologies and with activated cellular immune mechanisms. It is released from monocytes, macrophages, dendritic cells, and endothelial cells activated by interferon gamma (IFN-γ) secreted by Th1 lymphocytes [1,2]. There is a growing interest in this molecule as a diagnostic biomarker because its levels are elevated in some diseases (autoimmune, inflammatory, and malignant tumoral pathologies), and studies have reported increased levels in early phases of viral infections, in particular (e.g., EBV, CMV, and parvovirus B19) [3,4].

On the other hand, soluble CD14 (sCD14) is a glycosyl phosphatidyl inositolse (GPI) linked protein that is expressed as a receptor on the surface of cells such as monocyte/macrophages (M/Ms), polymorphonuclear leukocytes (PNLs), and dendritic cells involved in natural immune responses. [5]. When it is present in circulation along with mCD14, which is attached to the membrane and serves as a receptor for bacterial lipopolysaccharides (LPSs), it is released either on the surfaces of M/Ms with various stimuli or from intracellular pools, and it is an indicator for M/M activation [6,7]. It has been reported that sCD14 could be detected at high levels in pathologies such as trauma, sepsis, and rheumatoid arthritis and in patients with HBV, HCV, and HIV infections [5,8,9]. Increasing levels of sCD14 were shown to result from microbial translocation through the intestinal mucosa during HIV infection, and it is an independent predictor of mortality in HIV-infected patients [10,11].

Algorithms based on the verification of anti-HIV-1 scanning using enzyme immunoassays (EIAs) with WB tests were introduced in 1989 by The Center for Disease Control and Prevention (CDC) and The Association of Public Health Laboratories (APHL). These tests were frequently used until 2010 for HIV-1/2 diagnosis. In the USA and some Western countries, according to the M53-A guide by Clinical and Laboratory Standards Institute (CLSI), the ComboAg-Ab test with chemiluminescence micro-particle enzyme immunoassay (CMIA) and EIA could distinguish between HIV-1 and HIV-2. Tests based on nucleic acid amplification Technology (NAT) began to be used by the CDC in their algorithm in October 2014 [12–17].

The new algorithms replaced the relative instability of the previous algorithms in the USA and some Western countries. The older algorithms were noted for failure to diagnose HIV infection in early stages, misdiagnosis of acute HIV-1 infection, and difficulty in indeterminate HIV-1 (IHIV-1) diagnosis and its distinctiveness (it overlooked HIV-2). The IHIV-1 pattern was a serious problem in blood banks, risking the safety of the blood supply [15,17,18].

CD4 T-lymphocyte counts and molecular assays for quantifying plasma viral load are currently the standard methods used to monitor the immune activation status of patients with HIV infections. These methods require expensive equipment and experienced staff. Having alternative laboratory markers would reduce the cost of laboratory diagnosis and ease monitoring the immune activation status of those with HIV/AIDS in underdeveloped and developing countries [19,20]. Among efforts to improve new cost-effective methods are some that use biomarkers such as neopterin and sCD14, which indirectly assess the immune activation status of patients with HIV infections. In particular, plasma neopterin levels are reportedly correlated with plasma HIV viral load, but studies involved in measuring sCD14 levels are few in number [21]. Although neopterin and sCD14 studies have been performed using those with confirmed HIV-1 infections, no study involving those with IHIV-1 patterns can be found in the literature. Therefore, we aimed to evaluate the roles of the plasma immune activation biomarkers neopterin and soluble sCD14 in the indirect assessment of the immune activation status of patients with the IHIV-1 pattern and among a true HIV-1-positive infection (PCG).

## Material and Methods

### Study Area and Groups

This study was planned as cross-sectional study, and it was conducted as a multicenter study between October 2013 and June 2014. The centers involved in this study were:

1. the Serology/ELISA Laboratory of the Cerrahpasa Medical Faculty Medical Microbiology Department at Istanbul University,
2. the Turkish Red Crescent Marmara Region Blood Center Laboratory,
3. the Medical Microbiology Laboratory at the T.R. Health Ministry’s Skin and Reproduction Diseases Hospital in İstanbul’s Bakirköy Region, and
4. the Infectious Diseases Clinic at the T.R. Health Ministry’s Dr. Sadi Konuk Research and Training Hospital in Bakirköy.

In this study, 100 qualified people comprising the healthy control group (HCG) were compared to 88 patients with the IHIV-1 pattern and 100 patients with true positive HIV-1, the latter group comprising the positive control group (PCG). The sex (male/female) distribution and mean age among those with the IHIV-1 pattern, the PCG, and the HCG were, respectively, 76/12 and 37.4 years (range, 20–62 years); 81/19 and 41.4 years (range, 25–71 years); and 84/16 and 36.2 years (range, 18–60 years). Those with the IHIV-1 pattern and those in the PCG were matched with those in the HCG (*p* > 0.05).

Those with the IHIV-1 pattern and those in the PCG were examined according to CDC criteria. Serums were repeatedly tested using the anti-HIV-1 test (EIA method) and assayed for at least two of the three viral proteins (p24, gp41, and gp120/160) using the Western blot and LIA immunoassay (WB/LIA) tests for verification. If both were positive, the patient was assigned to the PCG. If none of the viral proteins were found, the patient was found negative for HIV, but if one of the proteins or one of the gag proteins (p17 or p55) or pol proteins (p66 or p31) or both were detected, the patient was found to have the IHIV-1 AIDS; HIV; sCD14; neopterin, indeterminate HIV-1 pattern [12].

#### IHIV-1 Pattern Group

To confirm IHIV-1, negative HBV and HCV tests (HBsAg and Anti-HCV) were performed six weeks later, and in those where the viral protein was found using WB/LIA, 30 were found with only p24 (24:±, 6:+), 22 were found with only gp41 (19:±, 3:+), 36 were found with a gag protein and/or a pol protein. One IHIV-1 patient with gp41 (+) was found to be positive for HIV-RNA, whereas all others were negative for HIV-RNA.

#### Patient Control Group

Among those in the PCG, all were positive for HIV-RNA. According to the 1993 revised classification by the CDC [12], 87 (87%) and 13 (13%) were B group with symptomatic non-AIDS and B group with asymptomatic HIV-1 infection, respectively.

#### Healthy Control Group

Those with negative HIV Ag/Ab, Anti-HCV, and HbsAg test results and without prior chronic disease in the previous year and without viral infections in the previous month were included in the HCG.

#### All Study Participants

All participants signed a written informed consent form approved by Clinical Research Ethics Board of Istanbul University, Cerrahpasa Faculty of Medicine (No:83045809/604, Date: 01.07.2014). These same Institutional Ethics Board also approved this study.

### Collection of Samples and Methods

We collected 10-ml blood samples without anticoagulant to determine neopterin and sCD14 levels and additional 10-ml blood samples from those in the PCG and with the IHIV-1 pattern with anticoagulant (EDTA) to determine HIV-RNA.

#### Immunological (Serological) Methods

The HIV Ab/Ag test was used as a screening test for HIV. The EIA/CMIA kits varied by center. At Istanbul University and the Infectious Diseases Clinic, the Genscreen Ultra HIV Ag-Ab test (Bio-Rad Laboratories, UK) was used. At the Turkish Red Cresent, the Liaison XL and Murex HIV Ab/Ag (Italy) was used. At the Skin and Reproductive Diseases Hospital, the HIV Ab/Ag Dia.Pro (Diagnostic Bioprobes, Italy) was used. Results were evaluated according to recommendations by the manufacturers, and when values were greater than the cut-off values, reactivity was recognized.

The LIA method via the immune-blot method was used to confirm recurrent reactive HIV Ab/Ag (Inno-LIA HIV-1/2 score; Innogenetics, Belgium) at all centers except the Skin and Reproductive Diseases Hospital, where the Western blot method test was used (HIV BLOT 2.2 assay MP; Biomedicals Asia Pasific Pte, Ltd, Singapore). Samples were evaluated according to recommendations by the manufacturers of the commercial LIA and WB kits. We considered CDC criteria as a basis for evaluation [12].

Except at the Turkish Red Cresent, the HbsAg test was performed using a commercial kit (Surase B-96; General Biologicals Corp., Taiwan) with a sandwich micro-ELISA method. Another commercial kit (NANBASE C-96 V4.0; General Biologicals Corp., Taiwan) with a sandwich micro-ELISA method for hepatitis C virus was used in a TRITURUS device, a direct open system (Diagnostics, Grifols, Spain). When values were above the cut-off value, reactivity was recognized.

At the Turkish Red Cresent, the HbsAg test was performed using Enzygnost (Siemens Healthcare Diagnostics Pproducts, Germany) with a sandwich micro-ELISA method. The anti-HCV test for hepatitis C virus, however, was utilized using a Monolisa Anti-HCV PLUS Version 2 kit (Bio-Rad, France) based on a micro-ELISA method. The Freedom Evolyzer 200 device (Tecan, Männedorf, Switzerland), which is fully automatic open system, was used in accordance with the recommendations of commercial kit (Tecan, Männedorf, Switzerland). When values were above the cut-off value, reactivity was recognized.

#### Molecular tests

To detect HIV-RNA, a Ampliprep/COBAS TagMan HIV-1 test v.2.0 (Roche, Switzerland) was used. Detection was as low as 20 copies/ml.

#### Neopterin and sCD14 tests related to the study

Serum samples were analyzed using a commercial neopterin test kit (DRG Instruments GMBH, Germany) in the TRITURUS device (Diagnostics, Grifols, Spain) using the quantitative competitive EIA method. A cut-off value of 13.245 nmol/L was calculated from receiver operating characteristic (ROC) curves for predicting HIV-1 infection. When levels were above this cut-off value, presence of infection was recognized.

Serum samples were analyzed for sCD14 using an Avizcera (Bioscience, USD) commercial kit in the TRITURUS device (Diagnostic, Grifols, Spain) using the quantitative sandwich EIA method. A cut-off value of 3.968 μg/ml was calculated from receiver operating characteristic (ROC) curves for predicting HIV-1 infection. When levels were above this cut-off value, presence of infection was recognized.

### Statistical Methods

Non-parametric Kruskal-Wallis One-way ANOVA with a post-hoc Tukey HSD test for multiple comparisons was used to compare subgroups for means of quantitative variables. A chi-square test was evaluated for categorical variables. Pearson correlation coefficients were calculated to characterize relationships between each quantitative variable within groups. The ROC curves were analyzed to determine diagnostic validity of the quantitative variables. All statistical analyses were calculated using IBM SPSS version 20 (IBM Corporation, Somers, NY) and MedCalc version 13 (MedCalc, Mariakerke, Belgium) statistical software packages. Signficance was recognized when *p* < 0.05.

Performance parameters of the neopterin and sCD14 tests (sensitivity, specificity, and positive and negative predictive values) and agreements between the diagnoses of HIV-1 infections were assessed using the kappa test. An agreement limit of at least 65% was used as the base value.

## Results

Baseline characteristics of all groups are shown in Table 1.

**Table 1:** Baseline Characteristics of Study and Control Groups.

Characteristics	Groups (n-%)		
	PCG (*N* = 100)	IHIV-1 (*N* = 88)	HCG (*N* = 100)
**Age (years)**			
Median(min-max)	41.4 (25–71)	37.4 (20–62)	36.2 (18–60)
**Sex**			
Male	81 (81%)	76 (86%)	84 (84%)
Female	19 (19%)	12 (14%)	16 (16%)
**Geographic Origin**			
Inside Istanbul	88 (88%)	80 (91%)	100 (100%)
Turkey, outside Istanbul	8 (8%)	6 (7%)	0 (0%)
Foreign national	4 (4%)	2 (2%)	0 (0%)
**Marital Status**			
Married	52 (52%)	46 (52%)	54 (54%)
Single	48 (48%)	42 (48%)	46 (46%)
**Education Level**			
Primary school	16 (16%)	16 (18%)	31 (31%)
High school	38 (38%)	36 (41%)	36 (36%)
University	46 (46%)	36 (41%)	33 (33%)
**HIV History**			
**CDC HIV Stage**		-	
Stage A	13(13%)		
Stage B (symptomatic non-AIDS)	87(87%)		
**Possible Ttransmission Route**			
Heterosexual	58 (58%)		-
Homosexual	26 (26%)		-
Unknown	16 (16%)		-
**CD4+ T-cell Count, Median**	609 (132–877)		
CD4+ T-cell count < 200 cells/μL	4 (4%)		-
CD4+ T-cell count 200–500 cells/μL	22 (22%)	-	
CD4+ T-cell count > 500 cells/μL	74 (74%)		-
Viral load <50 copies/ml	9 (9%)		-
**Tuberculosis History**			
Prior diagnosis of latent tbc infection	7 (7%)	1 (1%)	0 (0%)
History of BCG vaccination	75 (75%)	65 (%74)	80 (%80)
BCG vaccination status unknown	10 (10%)	9 (10%)	12 (12%)
**Other Viral Panel**			
HBV	0 (0%)	0 (0%)	0 (0%)
HCV	0 (0%)	0 (0%)	0 (0%)
**Clinical Application Causes**			
Blood donation		28 (32%)	
Preoperative		34 (38%)	
Routine check		26 (30%)	

Data are given as n (percent) or value (range).

**Fig 1:**
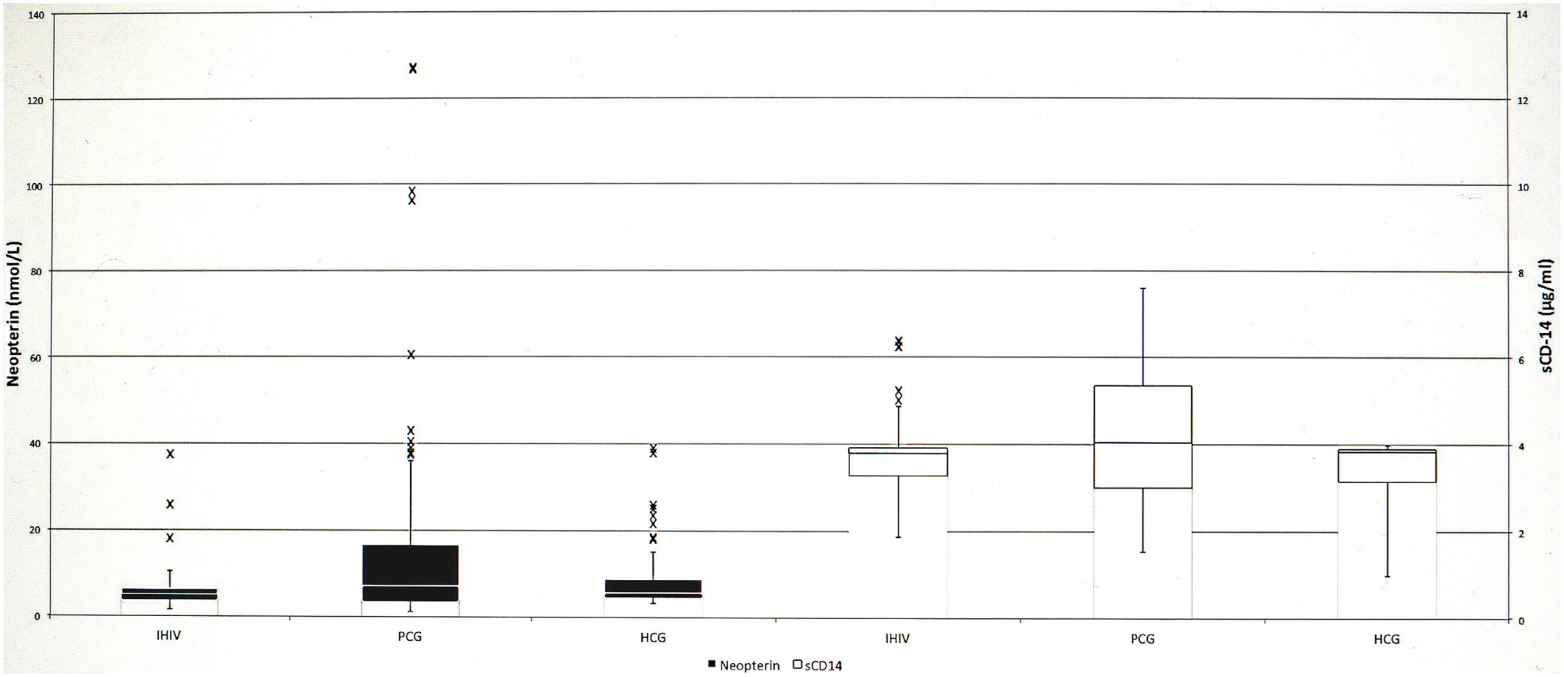
Side-by-side box-whisker plots of neopterin and sCD14evels in study and control groups when cut-off values 13.245 nmol/L and 3.968 μg/ml, respectively.

Neopterin and sCD14 levels among the PCG, HCG, and those with the IHIV-1 pattern are shown in Table 2. Mean neopterin and sCD14 levels among those with the IHIV-1 pattern were significantly lower than among those in the PCG (*p* < 0.001 and *p* = 0.001, respectively), but they were similiar to those in the HCG (*p* = 0.57 and *p* = 0.66, respectively). Nonetheless, mean neopterin and sCD14 levels among those in the PCG were significantly higher than those found among patients with the IHIV-1 pattern (*p* < 0.001 and *p* = 0.001, respectively) and those in the HCG (*p* = 0.001 and *p* < 0.001, respectively). See Fig 1.

**Table 2:** Comparison of Mean Neopterin and sCD14 Levels in Study and Control Groups.

Groups	Neopterin (nmol/L) Mean ± sd (min-max)	sCD14 (μg/ml) Mean ± sd (min-max)
IHIV-1 (*n* = 88)	5.73 ± 4.77 (1.58–38.71)	3.65 ± 0.79 (1.86–6.17)
PCG (*n* = 100)	15.77 ± 23.74 (1.19–127.2)	4.20 ± 1.51 (1.53–7.59)
HCG (*n* = 100)	7.90 ± 6.5 (3.15–39.3)	3.51 ± 0.54 (0.97–3.97)
**Statistical Value**		
IHIV-1 x PCG	*p* < 0.001	*p* = 0.001
IHIV-1 x HCG	*p* = 0.576	*p* = 0.66
PCG x HCG	*p* = 0.001	*p* < 0.001

IHIV-1: indeterminate HIV-1; PCG positive control group; HCG: healthy control group; sd: standard deviation.

**Fig 2:**
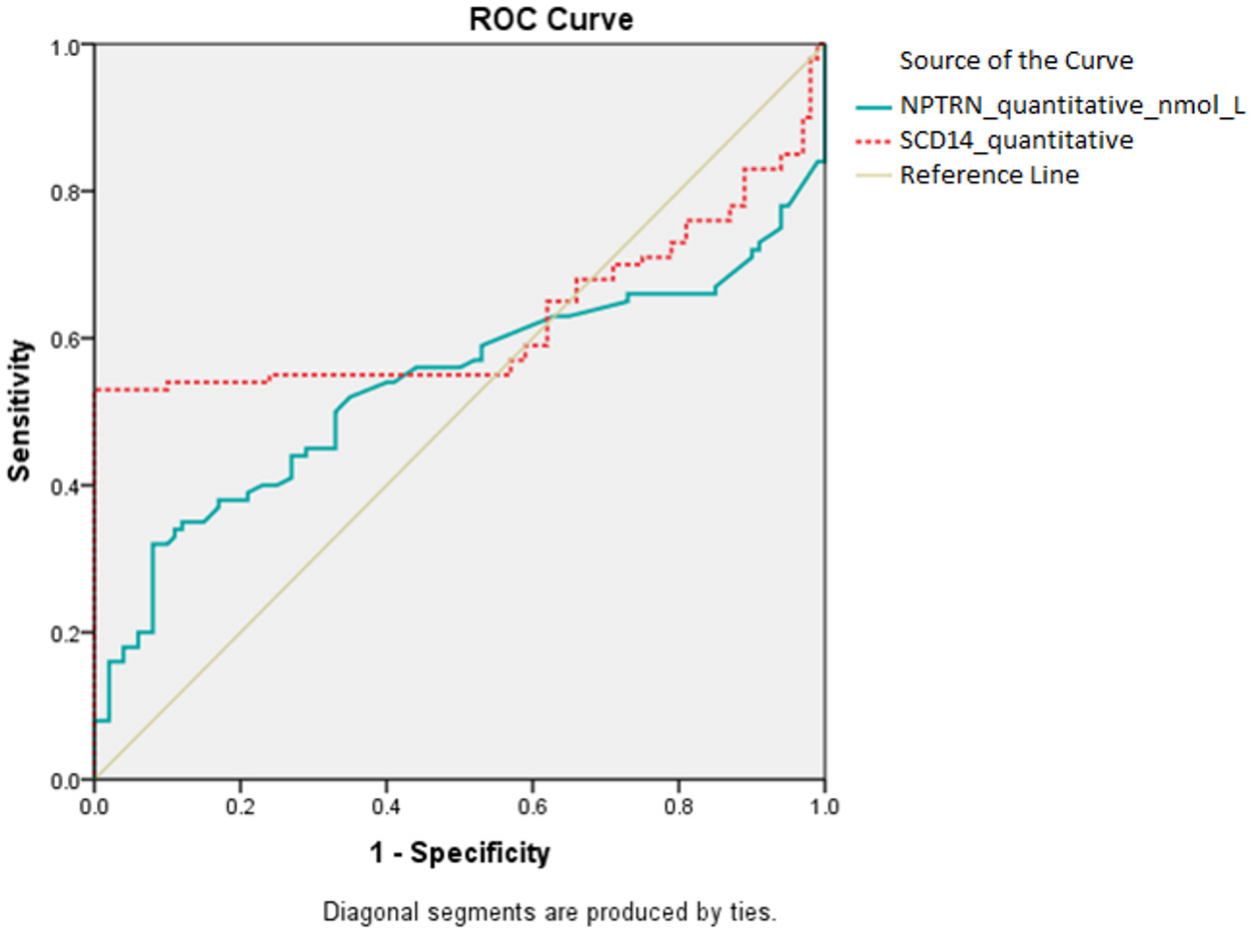
Receiver operating characteristic curves showing areas under the curve for neopterin and sCD14 for predicting HIV-1 infection. The neopterin cut-off value is 13.245 nmol/L, and the sCD14 cut-off value is 3.968 μg/ml.

Various sCD14 and neopterin cut-off values were examined for prediction of HIV-1 infection, and values of 3.968 μg/ml and 13.245 nmol/L resulted in the maximum area under the ROC curves, which were 0.627 (95% CI, 0.556–0.694; *p* = 0.0036) and 0.534 (95% CI, 0.463–0.605; *p* = 0.4256), respectively (Fig 2). These cut-offs corresponded to a sensitivity of 32% (32 of 100 patients) and a specificity of 92% (92 of 100 patients) for neopterin and of 53% (53 of 100 patients) and 100% (100 of 100 patients) for sCD14. While neopterin did not have an adequate predictive value for identifying true positive HIV-1 among the PCG (area under the curve [AUC] = 0.534; *p* = 0.4256), sCD14 did have an adequate predictive value (AUC = 0.627; *p* = 0.0036).

Results of ROC analyses defining the area under the receiver operating characteristic curves for neopterin and sCD14 are shown in Table 3.

**Table 3:** Diagnostic Performances of Neopterin and sCD14 in True Positive HIV-1 Patients.

	Cut-off value	AUC (95%CI)	Se. (%)	Spe. (%)	PPV	NPV	Kappa coefficient	*p value*
**Neopterin, nmol/L**	13.24	0.534(0.463–0.605)	32	92	78	91	25	*p* = 0.4256
**sCD14, μg/ml**	3.99	0.627 (0.556–0.694)	53	100	98	99	45	*p* = 0.0036

Se.: Sensitivity; Spe.: Specificity; AUC: area under the curve; PPV: positive predictive value; NPV: negative predictive value; CI: confidence interval.

## Discussion

The circulating levels of the plasma immune activation markers, including neopterin, sCD14, tumor necrosis factor receptor type II, and interleukin-2 receptors, increase in HIV-infected patients, and they represent immunologic changes throughout the body [22]. In recent years, there is growing interest in neopterin, which has been claimed to be very useful in the early diagnosis of viral infections such as HIV, CMV and parvovirus B19 as well as in donor scans for blood-borne infections at blood banks. Another marker, sCD14, is also of interest [4,5,23]. Increased neopterin levels have been indicated during acute viral infections with HIV, hepatitis viruses, Epstein-Barr virus, measles virus, mumps virus, varicella zoster virus, rubella virus, dengue virus, and influenza viruses. A correlation between neopterin and the activity of viral infections has also been observed. Neopterin is known to be produced by activated monocytes upon stimulation, mainly by interferon gamma and the detection of neopterin in high concentrations indicates the activation of the cellular immune system. In other words, it reflects the degree of immune activation [8,24]. IFN-gamma, in addition to having antiviral activity, has important immunoregulatory functions. Regarding IFN-gamma as an indicator of immune activation, the measured levels of neopterin and IFN-gamma were found to be similar in patients with HIV-1 and hepatitis C virus infections as well as those with hematological neoplasias [25,26,27]. Neopterin levels seem to reveal the total effect of the immunological network and interactions on the population of monocytes/macrophages [28]. In this respect, we may expect that neopterin levels have a potential to supply valuable information about the immune activation status of patients with HIV infections.

In the literature, there are some studies using neopterin as a prognostic marker for HIV infection, and most of these studies showed an increase in neopterin levels in people with HIV infection when compared to patients without HIV infection [22,29–31]. During viral infections, increased neopterin levels were correlated with disease activity and elevated neopterin levels were detected at the end of the incubation period and before the onset of clinical symptoms in most of the related studies. [8]. Highly active antiretroviral therapy (HAART) has shown to decrease neopterin levels significantly in HIV patients, and neopterin levels in patients who discontinued HAART were not affected [32]. It seems that excluding blood donors with high neopterin levels, those over a cut-off value of 10 nmol/l, would probably reduce the transmission risk of HIV infections in blood banks. In the first application of this hypothesis, since October 1986, all blood donors in the Tyrol state of Austria have been tested for neopterin levels. The 10-nmol/l cut-off was used as an exclusion criteria, and by doing so, 1.6% of 76,587 donors were excluded from the blood donation program. Causative agents responsible from the elevated neopterin levels were detected as viruses causing a viral respiratory tract infection in 123 (67%) cases. Six blood donors had toxoplasmosis, and 4 had HIV infection or non-A, non-B hepatitis [33]. In another study, Immanuel et al., [34] measured serum neopterin concentrations in HIV-seropositive and seronegative patients with tuberculosis before, during, and at the end of antituberculosis therapy. The HIV-infected asymptomatic individuals had serum neopterin levels that were higher than those of healthy controls but lower than those of HIV-TB patients. They concluded that neopterin levels decrease with anti-tuberculosis therapy; persistently elevated neopterin levels indicate a progressive HIV disease and a poor prognosis. In a review from Austria, neopterin was indicated as a reliable marker for HIV-1 infection, and it was suggested that neopterin synthesis occured at high levels prior to antibody seroconversion. After this stage, it was reported that neopterin concentration in urine and sera of individuals with HIV-1 infections were correlated with the viral load in circulation, and it was an important biomarker in displaying disease course, progression, and anti-retroviral treatment activity [35]. In a study carried out in Germany using 29 HIV-1 patients during a window period, high neopterin levels were found in the early phase of HIV-1 infection [36]. Yet in another study with similar results, the investigators concluded that neopterin is a beneficial marker in monitoring HIV treatment and progression of the disease [37]. Recently, Bipath et al., [38] concluded that plasma neopterin was a good indicator of inflammatory activity, inflammation-associated co-morbidities, and degree of immune deficiency in their study of 105 HIV/AIDS patients. They detected neopterin levels were significantly higher (*p* < 0.001) among the total patient group than in the control group, and significant correlations between neopterin and plasma indicators of inflammation showed neopterin to be a good indicator of active inflammatory status and of the effect of HAART on the immune system. Neopterin measurement is more cost-effective but less sensitive than screening using molecular-based assays. However, neopterin may be a useful biomarker for monitoring infectious disease activity indirectly, showing the immune activation status of patients during HIV treatment and predicting HIV infection progression [8]. Our neopterin results were in agreement with the previous studies. Mean levels of neopterin and sCD14 among those with the IHIV-1 pattern were significantly lower than those in the PCG, but they were similiar to those found in the HCG. Nonetheless, mean neopterin and sCD14 levels of among those with true positive HIV-1 were significantly higher than among those with the IHIV-1 pattern and those in the HCG. Analysis of ROC curves for the predicting HIV-1 infection using neopterin was not promising (RUC: 0.534) to use neopterin as a surrogate test. While the sensitivity of neopterin was very low (32%), specificity was high (92%) when 13.24 nmol/L was chosen as the cut-off value. We suggest that neopterin is not useful as a diagnostic marker, but its higher specificity (92%) may be partly useful for deciding whether a case is a true positive HIV-1 infection. Although most of our results were in good agreement with other studies, no reports are comparable to our results for those with the IHIV-1 pattern. Our normal neopterin levels found among those with the IHIV-1 pattern leads us to conclude that there was no evidence of a real HIV infection upon stimulation of monocytes by IFN-gamma released by T lymphocytes.

It is known that increased sCD14 released from monocytes/macrophages playing Trojan horse roles as the main reservoir cell group in HIV-1 infections is reportedly linked with monocytes’ continuous exposure to free gp120s or virus, according to *in vitro* cell experiences. Therefore, activation of monocytes/macrophages may result in both enhanced virus replication and an release in sCD14, which may further aggravate disease [9,38,39].

Another plausible explanation related to the sCD14 increase in HIV infections may be linked to the microbial translocation hypothesis. According to this hypothesis, levels of sCD14 increase as a result of microbial translocation through the intestinal mucosa during HIV infection [10]. sCD14 is a marker for monocyte/macrophage activation and a mediator of bacterial LPS action [40]. To support this idea, some cross-sectional studies showed a higher sCD14 level in HIV-infected people than in healthy people [41,42]. Romero-Sánchez et al. [43] also reported that the sCD14 levels, but not LPS, were independently related to progression of HIV disease, further supporting the clinical importance of sCD14. In a study conducted using 29 asymptomatic, 22 symptomatic non-AIDS, and 41 AIDS individuals by Lien et al., [44] showed that there was a marked increase in the soluble monocyte/macrophage activation marker sCD14 in all clinical stages of HIV-1 infection, with the highest levels in the AIDS group. Furthermore, sCD14 levels were significantly correlated with the degree of immunodeficiency and HIV-1 replication in their study. They suggested that sCD14 serum concentrations may reflect disease activity and viral load. Monitoring of monocyte/macrophage activity may therefore be of high clinical relevance in later stages of HIV-1 infection. Information is limited on the immunological and virological events that occur during the earliest stages of acute HIV infection when HIV serology is still non-reactive. The sCD14 level was found generally associated with mortality during the chronic phase of HIV infection. On the other hand, there is some evidence that sCD14 might also be produced during primary infection, earlier than other biomarkers of microbial translocation. Data indicate that, in acute HIV infection, T-cell and monocyte activation is closely dependent on viral replication and not on systemic microbial translocation that occurs later in the natural history of infection [45]. During acute HIV-1 infection, enhanced formation of neopterin occurs already at a very early time point, before antibody seroconversion takes place. Therefore, elevated urine or serum neopterin appears to be a very early marker of HIV infection and longitudinal studies have revealed that neopterin levels correlate with disease progression [29,35]. In our study, higher neopterin and sCD14 levels in the PCG than in the HCG is compatible with reports in the literature. Most of our cases (87%) with a true positive HIV-1 infection at stage B according to CDC-93 guideline, those who applied were not in an early (acute) phase. Meanwhile, other cases with (13%) at stage A had symptoms related to HIV-1 infection when they were included in the study. Therefore, it was not possible to predict mean neopterin and sCD14 levels before the onset of symptomatic HIV infection. Nonetheless, mean neopterin and sCD14 levels in those with the IHIV-1 pattern were close to those found in the HCG; there was no statistically significant difference between them. Also, detecting these markers in both groups (IHIV-1 pattern group and HCG) below the pathological cut-off levels and the lack of immune activation depending on an antigenic stimulation in these groups (INF-γ induction of neopterin based on Th1 or M/M activation with continous HIV/gp120 stimulation), suggest that this atypical pattern (IHIV-1) may not be an actual HIV-1 infection. Recombinant or natural glycoproteins such as virus-bound or circulating free viral antigens were shown to stimulate the release of inflammatory cytokines like sCD14 from monocytes [44]. ROC analysis of sCD14 for the prediction of HIV-1 infection was significant but sCD14 also had poor predictive value but high specificity (100%) for identifying those in the PCG (AUC = 0.627; 95% CI, 0.556–0.694; p = 0.0036). While the sensitivity of sCD14 was very low (53%), specificity was very high (100%) when 3.99 μg/ml was chosen as the cut-off value. We suggest that sCD14 is not useful as a diagnostic marker, but its high specificity (100%) may be useful for deciding whether a case is a true positive HIV-1 infection.

In addition, diagnostic performances of neopterin and sCD14 were evaluated as nonspecific tests, suggesting that although sensitivity and kappa coefficients were quite low (32% and 53%, respectively), specifity was high (92% and 100%, respectively). We suggested that sCD14 may be useful as the non-specific marker for indirect assessment of immune activation status among patients whether or not they have a true positive HIV-1 infection.

This study has some limitations. The diagnostic performances of neopterin and sCD14, particularly sensitivity and kappa coefficients, may have been affected. Because the majority (87%) of those in the PCG were at stage B (symptomatic non-AIDS) and the remainder were at stage A, according to CDC-93 guideline, those who applied were not in an early (acute) phase. Therefore, results for our PCG were consistent with the literature, indicating increased neopterin and sCD14 levels in infectious syndromes, such as HIV/AIDS, characterized by decreased cellular immun functions. Though neopterin and sCD14 results were reported for those with the IHIV-1 serological pattern for the first time, our results for these patients suggest that there was no viral-induction based immunoactivation.

In fact, improvements in prognostic specificity of HIV-1 can be gained by combining CD4 cell counts with neopterin and sCD14 data. In conclusion, obviously, these two markers cannot be used as sceening or diagnostic markers in diagnosing of HIV infections because they have low sensitivies. On the other hand, when the consequences of having a false positive test (for example, in the IHIV-1 pattern) are very serious (e.g., the psychological problems associated with falsely diagnosing HIV), a test with high specificity is also important. Our significant, but poor predictive results for sCD14 are derived from ROC analyses, suggesting that sCD14 may be also used to exclude IHIV-1 from HIV-1 infection possibilities. Taken together, we suggest that neopterin and sCD14 may be helpful because they both have high specificity (92%-100%) as indirect non-specific marker for predicting the immune activation status of individuals whether or not they have a true positive HIV-1 infection. New large-scale longitudinal studies are needed to clarify the value and practicality of monitoring these indirect measures of immune activation status as additional surrogate markers for patients with HIV infections.

## Supporting Information

S1 Fig ROC analysis of sCD14 for true positive HIV group (PCG). (DOCX)

S2 Fig ROC analysis of neopterin for true positive HIV group (PCG). (DOCX)

S1 Table Patient control group, neopterin and sCD14 mean values (n, 100). (XLSX)

S2 Table IHIV-1 group, neopterin and sCD14 mean values (n, 88). (XLSX)

S3 Table Healthy control group, neopterin and sCD14 mean values (n, 100). (XLSX)

S4 Table Correlations for all groups. (XLS)

S5 Table Results of One way innova and PosHoc test for comparing mean values of test parameters for all groups. (XLS)

S6 Table Sensitivities and specificities for patient control group (true positive HIV cases). (XLS)

S7 Table Calculation of Side-by-side box-whisker plots of neopterin and sCD14evels in study and control groups when cut-off values 13.245 nmol/L and 3.968 μg/ml, respectively. (XLSX)

S1 Text Sample size power analysis. (DOCX)

S2 Text Confirmative statistical analyses by Ahmet Dirican. (DOCX)
