# Supplementary material for: Neopterin and Soluble CD14 Levels as Indicators of Immune Activation in Cases with Indeterminate Pattern and True Positive HIV-1 Infection
Source: PLoS One. 2016 Mar 31;11(3):e0152258. doi: 10.1371/journal.pone.0152258 (PMC4816292; doi:10.1371/journal.pone.0152258)
Supplement: S1 Fig — (DOCX) [file pone.0152258.s001.docx]

HIV vs control

| Variable | SCD14 |
| --- | --- |
| Classification variable | hiv_vs_control |

| Sample size |  | 200 |
| --- | --- | --- |
| Positive group : | hiv_vs_control = 1 | 100 |
| Negative group : | hiv_vs_control = 0 | 100 |

| Disease prevalence (%) | unknown |
| --- | --- |

**Area under the ROC curve (AUC)**

| Area under the ROC curve (AUC) | 0.627 |
| --- | --- |
| Standard Error^a^ | 0.0436 |
| 95% Confidence interval^b^ | 0.556 to 0.694 |
| z statistic | 2.916 |
| Significance level P (Area=0.5) | 0.0036 |

İhiv vs control

| Variable | SCD14 |
| --- | --- |
| Classification variable | ihiv_vs_control |

| Sample size |  | 188 |
| --- | --- | --- |
| Positive group : | ihiv_vs_control = 1 | 88 |
| Negative group : | ihiv_vs_control = 0 | 100 |

| Disease prevalence (%) | unknown |
| --- | --- |

**Area under the ROC curve (AUC)**

| Area under the ROC curve (AUC) | 0.545 |
| --- | --- |
| Standard Error^a^ | 0.0433 |
| 95% Confidence interval^b^ | 0.471 to 0.618 |
| z statistic | 1.042 |
| Significance level P (Area=0.5) | 0.2975 |

Total hiv + ihiv vs control

| Variable | SCD14 |
| --- | --- |
| Classification variable | totalhiv_vs_control |

| Sample size |  | 288 |
| --- | --- | --- |
| Positive group : | totalhiv_vs_control = 1 | 188 |
| Negative group : | totalhiv_vs_control = 0 | 100 |

| Disease prevalence (%) | unknown |
| --- | --- |

**Area under the ROC curve (AUC)**

| Area under the ROC curve (AUC) | 0.589 |
| --- | --- |
| Standard Error^a^ | 0.0329 |
| 95% Confidence interval^b^ | 0.530 to 0.646 |
| z statistic | 2.699 |
| Significance level P (Area=0.5) | 0.0070 |
