# Supplementary material for: Neopterin and Soluble CD14 Levels as Indicators of Immune Activation in Cases with Indeterminate Pattern and True Positive HIV-1 Infection
Source: PLoS One. 2016 Mar 31;11(3):e0152258. doi: 10.1371/journal.pone.0152258 (PMC4816292; doi:10.1371/journal.pone.0152258)
Supplement: S2 Fig — (DOCX) [file pone.0152258.s002.docx]

HIV vs control

| Variable 1 | SCD14 |
| --- | --- |
| Variable 2 | NPTRN_ngml |
| Classification variable | hiv_vs_control |

| Sample size |  | 200 |
| --- | --- | --- |
| Positive group : | hiv_vs_control = 1 | 100 |
| Negative group : | hiv_vs_control = 0 | 100 |

|  | AUC | SE ^a^ | 95% CI ^b^ |
| --- | --- | --- | --- |
| SCD14 | 0.627 | 0.0436 | 0.556 to 0.694 |
| NPTRN_ngml | 0.534 | 0.0431 | 0.463 to 0.605 |

^a^ DeLong et al., 1988

^b^ Binomial exact

**Pairwise comparison of ROC curves**

| SCD14 ~ NPTRN_ngml | |
| --- | --- |
| Difference between areas | 0.0928 |
| Standard Error^c^ | 0.0574 |
| 95% Confidence Interval | -0.0196 to 0.205 |
| z statistic | 1.619 |
| Significance level | P = 0.1055 |

No significant diffference between two AUCs of neopterin and sCD14

**only NPTRN ROC analysis**

| Variable | NPTRN_ngml |
| --- | --- |
| Classification variable | hiv_vs_control |

| Sample size |  | 200 |
| --- | --- | --- |
| Positive group : | hiv_vs_control = 1 | 100 |
| Negative group : | hiv_vs_control = 0 | 100 |

| Disease prevalence (%) | unknown |
| --- | --- |

**Area under the ROC curve (AUC)**

| Area under the ROC curve (AUC) | 0.534 |
| --- | --- |
| Standard Error^a^ | 0.0431 |
| 95% Confidence interval^b^ | 0.463 to 0.605 |
| z statistic | 0.797 |
| Significance level P (Area=0.5) | 0.4256 |

Total HIV vs control

| Variable 1 | NPTRN_ngml |
| --- | --- |
| Variable 2 | SCD14 |
| Classification variable | totalhiv_vs_control |

| Sample size |  | 288 |
| --- | --- | --- |
| Positive group : | totalhiv_vs_control = 1 | 188 |
| Negative group : | totalhiv_vs_control = 0 | 100 |

|  | AUC | SE ^a^ | 95% CI ^b^ |
| --- | --- | --- | --- |
| NPTRN_ngml | 0.546 | 0.0337 | 0.486 to 0.604 |
| SCD14 | 0.589 | 0.0329 | 0.530 to 0.646 |

^a^ DeLong et al., 1988

^b^ Binomial exact

**Pairwise comparison of ROC curves**

| NPTRN_ngml ~ SCD14 | |
| --- | --- |
| Difference between areas | 0.0432 |
| Standard Error^c^ | 0.0501 |
| 95% Confidence Interval | -0.0550 to 0.142 |
| z statistic | 0.863 |
| Significance level | P = 0.3884 |
