# Supplementary material for: Neopterin and Soluble CD14 Levels as Indicators of Immune Activation in Cases with Indeterminate Pattern and True Positive HIV-1 Infection
Source: PLoS One. 2016 Mar 31;11(3):e0152258. doi: 10.1371/journal.pone.0152258 (PMC4816292; doi:10.1371/journal.pone.0152258)
Supplement: S1 Text — (DOCX) [file pone.0152258.s010.docx]

Sample size : Power Analysis


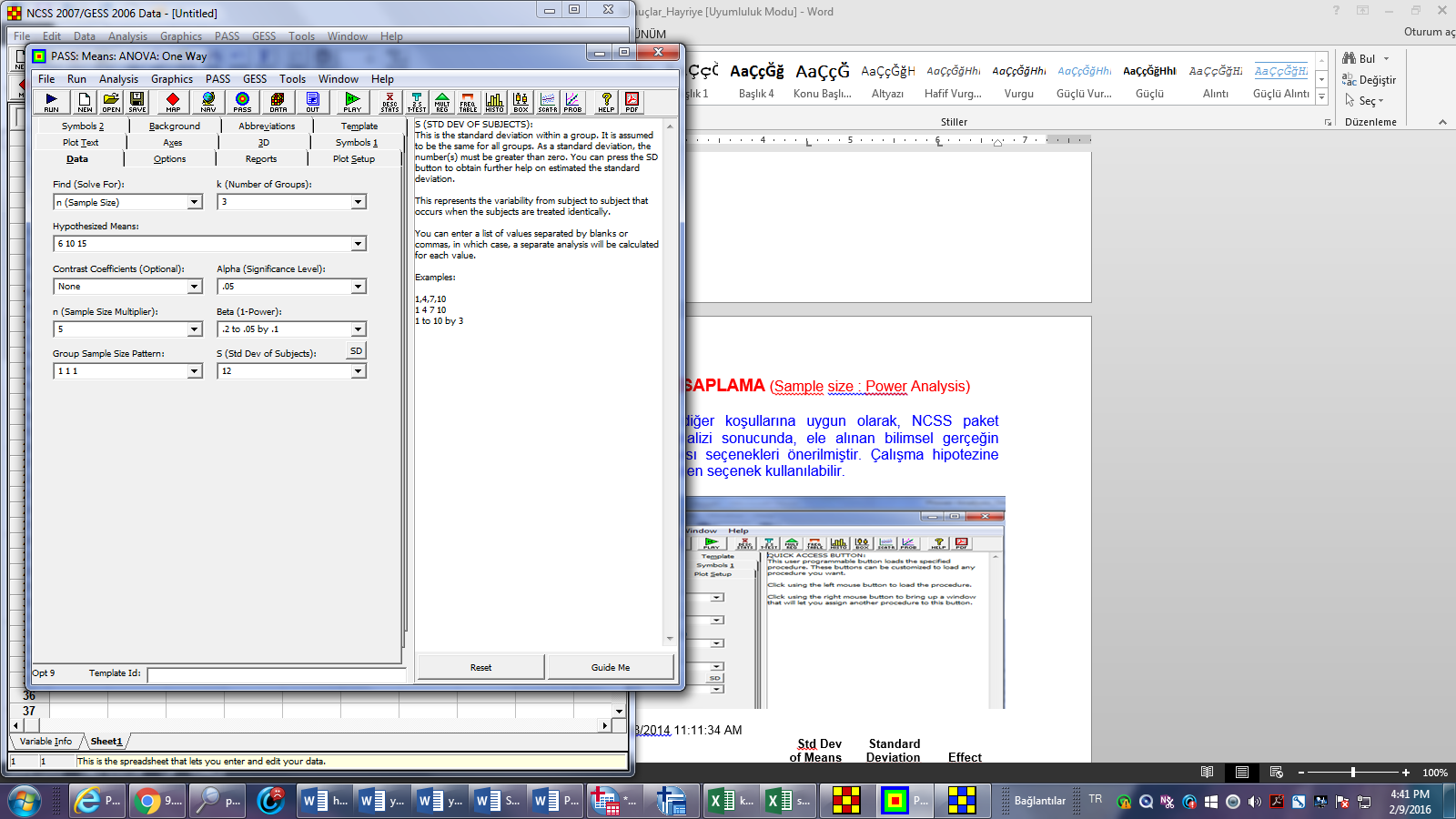


**One Way ANOVA Power Analysis**

Page/Date/Time 1 2/9/2016 4:22:04 PM

**Numeric Results**

**Std Dev Standard**

**Average Total of Means Deviation Effect**

**Power n k N Alpha Beta (Sm) (S) Size**

**0.95109 56.00 3 168 0.05000 0.04891 3.68 12.00 0.3068**

0.85292 40.00 3 120 0.05000 0.14708 3.68 12.00 0.3068

**References**

Desu, M. M. and Raghavarao, D. 1990. Sample Size Methodology. Academic Press. New York.

Fleiss, Joseph L. 1986. The Design and Analysis of Clinical Experiments. John Wiley & Sons. New York.

Kirk, Roger E. 1982. Experimental Design: Procedures for the Behavioral Sciences. Brooks/Cole. Pacific Grove,

California.

**Report Definitions**

Power is the probability of rejecting a false null hypothesis. It should be close to one.

n is the average group sample size.

k is the number of groups.

Total N is the total sample size of all groups combined.

Alpha is the probability of rejecting a true null hypothesis. It should be small.

Beta is the probability of accepting a false null hypothesis. It should be small.

Sm is the standard deviation of the group means under the alternative hypothesis.

Standard deviation is the within group standard deviation.

The Effect Size is the ratio of Sm to standard deviation.

**Summary Statements**

In a one-way ANOVA study, sample sizes of 56, 56, and 56 are obtained from the 3 groups whose

means are to be compared. The total sample of 168 subjects achieves 95% power to detect

differences among the means versus the alternative of equal means using an F test with a

0.05000 significance level. The size of the variation in the means is represented by their

standard deviation which is 3.68. The common standard deviation within a group is assumed to be

12.00.

**Details when Alpha = 0.05000, Power = 0.95109, SM = 3.68, S = 12.00**

**Percent Deviation Ni**

**Ni of From Times**

**Group Ni Total Ni Mean Mean Deviation**

1 56 33.33 6.00 4.33 242.67

2 56 33.33 10.00 0.33 18.67

3 56 33.33 15.00 4.67 261.33

ALL 168 100.00 10.33

As a result of power analysis for neopterin in three groups for One Way ANOVA Power Analysis, Alpha sinificance level calculated as o.o5, test power was calculated as 95% (Beta was calculated as 0.05), standart deviation was calculated as 12 and effect size was calculated as 0.3068. As conclusion, at least 56 cases were needed for each group. Our groups were 100,100 and 88 for patient control, IHIV (PCG) and healthy control groups, respectively. We added the calculation of power analyses to the supplemental section at the end of manuscript.

Prof. Dr. Ahmet Dirican
