# Supplementary material for: Neopterin and Soluble CD14 Levels as Indicators of Immune Activation in Cases with Indeterminate Pattern and True Positive HIV-1 Infection
Source: PLoS One. 2016 Mar 31;11(3):e0152258. doi: 10.1371/journal.pone.0152258 (PMC4816292; doi:10.1371/journal.pone.0152258)
Supplement: S2 Text — (DOCX) [file pone.0152258.s011.docx]

**ROC Curve**

[DataSet1]

| **Case Processing Summary** | |
| --- | --- |
| H1_K0 | Valid N (listwise) |
| Positive^a^ | 100 |
| Negative | 100 |
| Missing | 88 |

| Larger values of the test result variable(s) indicate stronger evidence for a positive actual state. |
| --- |
| a. The positive actual state is 1. |


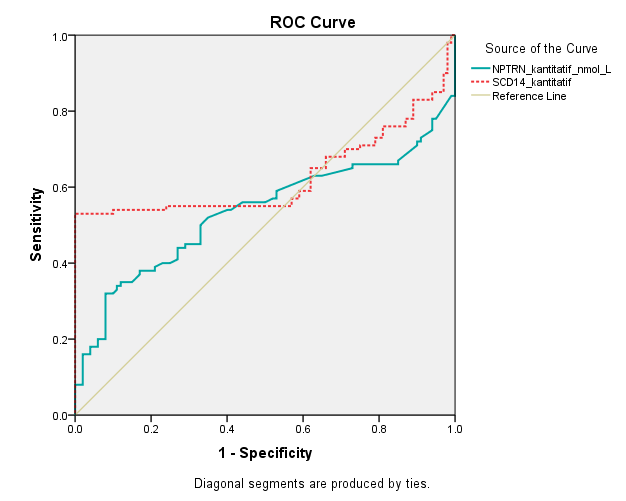


| **Area Under the Curve** | | | | | |
| --- | --- | --- | --- | --- | --- |
| Test Result Variable(s) | Area | Std. Error^a^ | Asymptotic Sig.^b^ | Asymptotic 95% Confidence Interval | |
|  |  |  |  | Lower Bound | Upper Bound |
| NPTRN_kantitatif_nmol_L | .534 | .043 | .401 | .450 | .619 |
| SCD14_kantitatif | .627 | .043 | .002 | .542 | .712 |

| The test result variable(s): NPTRN_kantitatif_nmol_L has at least one tie between the positive actual state group and the negative actual state group. Statistics may be biased. |
| --- |
| a. Under the nonparametric assumption |
| b. Null hypothesis: true area = 0.5 |

| **Coordinates of the Curve** | | | |
| --- | --- | --- | --- |
| Test Result Variable(s) | Positive if Greater Than or Equal To^a^ | Sensitivity | 1 - Specificity |
| NPTRN_kantitatif_nmol_L | .1850 | 1.000 | 1.000 |
|  | 1.1900 | .990 | 1.000 |
|  | 1.3875 | .980 | 1.000 |
|  | 1.6850 | .960 | 1.000 |
|  | 1.8825 | .940 | 1.000 |
|  | 2.1225 | .920 | 1.000 |
|  | 2.3200 | .910 | 1.000 |
|  | 2.3800 | .890 | 1.000 |
|  | 2.4775 | .880 | 1.000 |
|  | 2.6650 | .870 | 1.000 |
|  | 2.9575 | .840 | 1.000 |
|  | 3.1550 | .840 | .990 |
|  | 3.1700 | .780 | .950 |
|  | 3.3425 | .780 | .940 |
|  | 3.5175 | .770 | .940 |
|  | 3.5400 | .760 | .940 |
|  | 3.5525 | .750 | .940 |
|  | 3.5750 | .730 | .910 |
|  | 3.6675 | .720 | .910 |
|  | 3.7475 | .720 | .900 |
|  | 3.8525 | .710 | .900 |
|  | 4.1225 | .670 | .850 |
|  | 4.3200 | .660 | .850 |
|  | 4.3450 | .660 | .830 |
|  | 4.4375 | .660 | .770 |
|  | 4.5425 | .660 | .760 |
|  | 4.5850 | .660 | .730 |
|  | 4.6775 | .650 | .730 |
|  | 4.8325 | .630 | .650 |
|  | 4.9375 | .630 | .640 |
|  | 5.0425 | .630 | .630 |
|  | 5.1350 | .630 | .630 |
|  | 5.1475 | .590 | .530 |
|  | 5.2400 | .570 | .530 |
|  | 5.4250 | .570 | .520 |
|  | 5.6350 | .560 | .500 |
|  | 5.8325 | .560 | .490 |
|  | 6.0300 | .560 | .460 |
|  | 6.2275 | .560 | .440 |
|  | 6.4250 | .540 | .410 |
|  | 6.6225 | .540 | .400 |
|  | 6.9125 | .520 | .350 |
|  | 7.1300 | .500 | .330 |
|  | 7.3275 | .490 | .330 |
|  | 7.5925 | .460 | .330 |
|  | 7.6850 | .450 | .330 |
|  | 7.7025 | .450 | .320 |
|  | 7.8075 | .450 | .310 |
|  | 7.9450 | .450 | .290 |
|  | 8.0375 | .440 | .290 |
|  | 8.1880 | .440 | .270 |
|  | 8.2930 | .430 | .270 |
|  | 8.4925 | .410 | .270 |
|  | 8.7825 | .400 | .250 |
|  | 8.9800 | .400 | .230 |
|  | 9.0850 | .390 | .210 |
|  | 9.3875 | .380 | .210 |
|  | 9.7825 | .380 | .200 |
|  | 9.9675 | .380 | .180 |
|  | 10.1025 | .380 | .170 |
|  | 10.4050 | .370 | .170 |
|  | 10.8625 | .350 | .150 |
|  | 11.2575 | .350 | .120 |
|  | 11.5475 | .340 | .120 |
|  | 11.7325 | .340 | .110 |
|  | 12.0350 | .330 | .110 |
|  | 12.4425 | .320 | .100 |
|  | 12.9425 | .320 | .090 |
|  | 13.5350 | .320 | .080 |
|  | 13.8350 | .310 | .080 |
|  | 14.0950 | .300 | .080 |
|  | 14.4800 | .290 | .080 |
|  | 15.0100 | .280 | .080 |
|  | 15.5400 | .270 | .080 |
|  | 16.1225 | .260 | .080 |
|  | 16.5800 | .250 | .080 |
|  | 16.6825 | .240 | .080 |
|  | 16.9425 | .230 | .080 |
|  | 17.8375 | .220 | .080 |
|  | 18.6475 | .210 | .080 |
|  | 18.8450 | .200 | .080 |
|  | 19.5525 | .200 | .060 |
|  | 20.8000 | .190 | .060 |
|  | 21.8800 | .180 | .060 |
|  | 22.8050 | .180 | .050 |
|  | 24.3550 | .180 | .040 |
|  | 25.5400 | .170 | .040 |
|  | 26.2675 | .160 | .040 |
|  | 28.4400 | .160 | .020 |
|  | 30.5250 | .150 | .020 |
|  | 31.6475 | .140 | .020 |
|  | 33.3150 | .130 | .020 |
|  | 34.5625 | .120 | .020 |
|  | 36.1425 | .110 | .020 |
|  | 38.1425 | .090 | .020 |
|  | 38.8850 | .080 | .020 |
|  | 39.1550 | .080 | .010 |
|  | 39.9825 | .080 | .000 |
|  | 42.2550 | .070 | .000 |
|  | 52.2550 | .060 | .000 |
|  | 64.6975 | .050 | .000 |
|  | 82.7525 | .040 | .000 |
|  | 97.6700 | .030 | .000 |
|  | 112.8575 | .020 | .000 |
|  | 127.1700 | .010 | .000 |
|  | 128.1900 | .000 | .000 |
| SCD14_kantitatif | -.03000000 | 1.000 | 1.000 |
|  | 1.25100400 | 1.000 | .990 |
|  | 1.62470400 | .990 | .990 |
|  | 1.73370000 | .980 | .990 |
|  | 1.75910400 | .980 | .980 |
|  | 1.76820800 | .980 | .980 |
|  | 1.89901600 | .960 | .980 |
|  | 2.08919200 | .940 | .980 |
|  | 2.18227200 | .930 | .980 |
|  | 2.24291600 | .920 | .980 |
|  | 2.27992400 | .900 | .980 |
|  | 2.33156000 | .900 | .970 |
|  | 2.38154000 | .890 | .970 |
|  | 2.41303200 | .880 | .970 |
|  | 2.46432400 | .870 | .970 |
|  | 2.51227600 | .860 | .970 |
|  | 2.57100400 | .850 | .970 |
|  | 2.61500000 | .850 | .960 |
|  | 2.66735600 | .850 | .940 |
|  | 2.71605600 | .840 | .940 |
|  | 2.73370000 | .830 | .940 |
|  | 2.79000000 | .830 | .910 |
|  | 2.86000000 | .830 | .900 |
|  | 2.89783600 | .830 | .890 |
|  | 2.91056000 | .810 | .890 |
|  | 2.91618400 | .800 | .890 |
|  | 2.92346000 | .780 | .890 |
|  | 2.93700000 | .780 | .870 |
|  | 2.95200000 | .760 | .870 |
|  | 2.96250000 | .760 | .860 |
|  | 2.96750000 | .760 | .850 |
|  | 2.97500000 | .760 | .840 |
|  | 2.98500000 | .760 | .820 |
|  | 2.99082400 | .760 | .810 |
|  | 2.99962800 | .750 | .810 |
|  | 3.00807600 | .740 | .810 |
|  | 3.02427200 | .730 | .810 |
|  | 3.04326400 | .730 | .790 |
|  | 3.08326400 | .710 | .790 |
|  | 3.13000000 | .710 | .770 |
|  | 3.14428000 | .710 | .750 |
|  | 3.14928000 | .700 | .750 |
|  | 3.17500000 | .700 | .730 |
|  | 3.20809200 | .700 | .710 |
|  | 3.21809200 | .680 | .710 |
|  | 3.25500000 | .680 | .690 |
|  | 3.30000000 | .680 | .680 |
|  | 3.31641200 | .680 | .660 |
|  | 3.34797200 | .670 | .660 |
|  | 3.38154000 | .660 | .660 |
|  | 3.38998000 | .650 | .660 |
|  | 3.39500000 | .650 | .640 |
|  | 3.40261600 | .650 | .620 |
|  | 3.41031200 | .640 | .620 |
|  | 3.42574800 | .630 | .620 |
|  | 3.46113200 | .620 | .620 |
|  | 3.48935200 | .610 | .620 |
|  | 3.54728400 | .600 | .620 |
|  | 3.60601200 | .590 | .620 |
|  | 3.62000000 | .590 | .610 |
|  | 3.64000000 | .590 | .600 |
|  | 3.66730000 | .590 | .590 |
|  | 3.70230000 | .570 | .590 |
|  | 3.73494800 | .570 | .570 |
|  | 3.74994800 | .560 | .570 |
|  | 3.78500000 | .550 | .570 |
|  | 3.82500000 | .550 | .550 |
|  | 3.83500000 | .550 | .540 |
|  | 3.84500000 | .550 | .480 |
|  | 3.85500000 | .550 | .460 |
|  | 3.86500000 | .550 | .380 |
|  | 3.87250000 | .550 | .340 |
|  | 3.87750000 | .550 | .320 |
|  | 3.88250000 | .550 | .280 |
|  | 3.88650000 | .550 | .260 |
|  | 3.88888400 | .550 | .240 |
|  | 3.88988400 | .540 | .240 |
|  | 3.89500000 | .540 | .170 |
|  | 3.90500000 | .540 | .120 |
|  | 3.91272400 | .540 | .100 |
|  | 3.91772400 | .530 | .100 |
|  | 3.93000000 | .530 | .090 |
|  | 3.95000000 | .530 | .050 |
|  | 3.96250000 | .530 | .040 |
|  | 3.96650000 | .530 | .030 |
|  | 3.97900000 | .530 | .010 |
|  | 3.99082400 | .530 | .000 |
|  | 3.99171600 | .520 | .000 |
|  | 4.00016400 | .510 | .000 |
|  | 4.05296400 | .500 | .000 |
|  | 4.17686800 | .480 | .000 |
|  | 4.26304000 | .460 | .000 |
|  | 4.29627600 | .440 | .000 |
|  | 4.33524800 | .430 | .000 |
|  | 4.37881200 | .420 | .000 |
|  | 4.41267200 | .410 | .000 |
|  | 4.46869200 | .380 | .000 |
|  | 4.54028000 | .370 | .000 |
|  | 4.58029600 | .360 | .000 |
|  | 4.62739200 | .350 | .000 |
|  | 4.67472000 | .340 | .000 |
|  | 4.79322400 | .330 | .000 |
|  | 4.94077600 | .320 | .000 |
|  | 4.99969600 | .310 | .000 |
|  | 5.02856800 | .300 | .000 |
|  | 5.06834800 | .290 | .000 |
|  | 5.10607200 | .280 | .000 |
|  | 5.23632400 | .260 | .000 |
|  | 5.37645200 | .250 | .000 |
|  | 5.40759200 | .240 | .000 |
|  | 5.44805600 | .230 | .000 |
|  | 5.50407600 | .220 | .000 |
|  | 5.54028000 | .210 | .000 |
|  | 5.60566400 | .200 | .000 |
|  | 5.67472000 | .190 | .000 |
|  | 5.79019200 | .180 | .000 |
|  | 5.95560400 | .160 | .000 |
|  | 6.03851600 | .140 | .000 |
|  | 6.13275600 | .130 | .000 |
|  | 6.32936800 | .120 | .000 |
|  | 6.50631600 | .100 | .000 |
|  | 6.64229600 | .080 | .000 |
|  | 6.86179600 | .050 | .000 |
|  | 7.04802400 | .030 | .000 |
|  | 7.33889200 | .020 | .000 |
|  | 8.59061600 | .000 | .000 |

| The test result variable(s): NPTRN_kantitatif_nmol_L has at least one tie between the positive actual state group and the negative actual state group. |
| --- |
| a. The smallest cutoff value is the minimum observed test value minus 1, and the largest cutoff value is the maximum observed test value plus 1. All the other cutoff values are the averages of two consecutive ordered observed test values. |

**Oneway**

[DataSet1]

| **Descriptives** | | | | | | | | | |
| --- | --- | --- | --- | --- | --- | --- | --- | --- | --- |
|  | | N | Mean | Std. Deviation | Std. Error | 95% Confidence Interval for Mean | | Minimum | Maximum |
|  |  |  |  |  |  | Lower Bound | Upper Bound |  |  |
| NPTRN_kantitatif_nmol_L | 0 | 100 | 7.9069 | 6.49619 | .64962 | 6.6179 | 9.1959 | 3.15 | 39.30 |
|  | 1 | 100 | 15.7685 | 23.74121 | 2.37412 | 11.0577 | 20.4792 | 1.19 | 127.19 |
|  | 2 | 88 | 5.7366 | 4.76992 | .50847 | 4.7259 | 6.7472 | 1.58 | 38.71 |
|  | Total | 288 | 9.9735 | 15.31585 | .90250 | 8.1971 | 11.7498 | 1.19 | 127.19 |
| SCD14_kantitatif | 0 | 100 | 3.51562000 | .536303167 | .053630317 | 3.40920582 | 3.62203418 | .970000 | 3.990000 |
|  | 1 | 100 | 4.20629512 | 1.507754472 | .150775447 | 3.90712392 | 4.50546632 | 1.532008 | 7.590616 |
|  | 2 | 88 | 3.64832618 | .791458118 | .084369719 | 3.48063223 | 3.81602013 | 1.863608 | 6.166584 |
|  | Total | 288 | 3.79598686 | 1.079820292 | .063629021 | 3.67074814 | 3.92122558 | .970000 | 7.590616 |

| **Test of Homogeneity of Variances** | | | | |
| --- | --- | --- | --- | --- |
|  | Levene Statistic | df1 | df2 | Sig. |
| NPTRN_kantitatif_nmol_L | 31.562 | 2 | 285 | .000 |
| SCD14_kantitatif | 51.065 | 2 | 285 | .000 |

| **ANOVA** | | | | | | |
| --- | --- | --- | --- | --- | --- | --- |
|  | | Sum of Squares | df | Mean Square | F | Sig. |
| NPTRN_kantitatif_nmol_L | Between Groups | 5364.963 | 2 | 2682.481 | 12.339 | .000 |
|  | Within Groups | 61958.124 | 285 | 217.397 |  |  |
|  | Total | 67323.087 | 287 |  |  |  |
| SCD14_kantitatif | Between Groups | 26.615 | 2 | 13.307 | 12.312 | .000 |
|  | Within Groups | 308.031 | 285 | 1.081 |  |  |
|  | Total | 334.645 | 287 |  |  |  |

**Post Hoc Tests**

| **Multiple Comparisons** | | | | | | | | |
| --- | --- | --- | --- | --- | --- | --- | --- | --- |
| Dependent Variable | | (I) H1_K0 | (J) H1_K0 | Mean Difference (I-J) | Std. Error | Sig. | 95% Confidence Interval | |
|  |  |  |  |  |  |  | Lower Bound | Upper Bound |
| NPTRN_kantitatif_nmol_L | Tukey HSD | 0 | 1 | -7.86156^*^ | 2.08517 | .001 | -12.7743 | -2.9488 |
|  |  |  | 2 | 2.17031 | 2.15508 | .573 | -2.9071 | 7.2477 |
|  |  | 1 | 0 | 7.86156^*^ | 2.08517 | .001 | 2.9488 | 12.7743 |
|  |  |  | 2 | 10.03187^*^ | 2.15508 | .000 | 4.9544 | 15.1093 |
|  |  | 2 | 0 | -2.17031 | 2.15508 | .573 | -7.2477 | 2.9071 |
|  |  |  | 1 | -10.03187^*^ | 2.15508 | .000 | -15.1093 | -4.9544 |
|  | Dunnett t (2-sided)^b^ | 1 | 0 | 7.86156^*^ | 2.08517 | .000 | 3.2222 | 12.5010 |
|  |  | 2 | 0 | -2.17031 | 2.15508 | .500 | -6.9653 | 2.6246 |
| SCD14_kantitatif | Tukey HSD | 0 | 1 | -.690675120^*^ | .147024485 | .000 | -1.03706846 | -.34428178 |
|  |  |  | 2 | -.132706182 | .151954042 | .658 | -.49071368 | .22530132 |
|  |  | 1 | 0 | .690675120^*^ | .147024485 | .000 | .34428178 | 1.03706846 |
|  |  |  | 2 | .557968938^*^ | .151954042 | .001 | .19996144 | .91597644 |
|  |  | 2 | 0 | .132706182 | .151954042 | .658 | -.22530132 | .49071368 |
|  |  |  | 1 | -.557968938^*^ | .151954042 | .001 | -.91597644 | -.19996144 |
|  | Dunnett t (2-sided)^b^ | 1 | 0 | .690675120^*^ | .147024485 | .000 | .36355358 | 1.01779666 |
|  |  | 2 | 0 | .132706182 | .151954042 | .590 | -.20538335 | .47079572 |

| *. The mean difference is significant at the 0.05 level. |
| --- |
| b. Dunnett t-tests treat one group as a control, and compare all other groups against it. |

**Homogeneous Subsets**

| **NPTRN_kantitatif_nmol_L** | | | | |
| --- | --- | --- | --- | --- |
|  | H1_K0 | N | Subset for alpha = 0.05 | |
|  |  |  | 1 | 2 |
| Tukey HSD^a,b^ | 2 | 88 | 5.7366 |  |
|  | 0 | 100 | 7.9069 |  |
|  | 1 | 100 |  | 15.7685 |
|  | Sig. |  | .566 | 1.000 |

| Means for groups in homogeneous subsets are displayed. |
| --- |
| a. Uses Harmonic Mean Sample Size = 95.652. |
| b. The group sizes are unequal. The harmonic mean of the group sizes is used. Type I error levels are not guaranteed. |

| **SCD14_kantitatif** | | | | |
| --- | --- | --- | --- | --- |
|  | H1_K0 | N | Subset for alpha = 0.05 | |
|  |  |  | 1 | 2 |
| Tukey HSD^a,b^ | 0 | 100 | 3.51562000 |  |
|  | 2 | 88 | 3.64832618 |  |
|  | 1 | 100 |  | 4.20629512 |
|  | Sig. |  | .652 | 1.000 |

| Means for groups in homogeneous subsets are displayed. |
| --- |
| a. Uses Harmonic Mean Sample Size = 95.652. |
| b. The group sizes are unequal. The harmonic mean of the group sizes is used. Type I error levels are not guaranteed. |

**Nonparametric Tests**

[DataSet1]


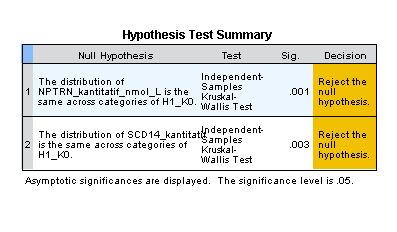


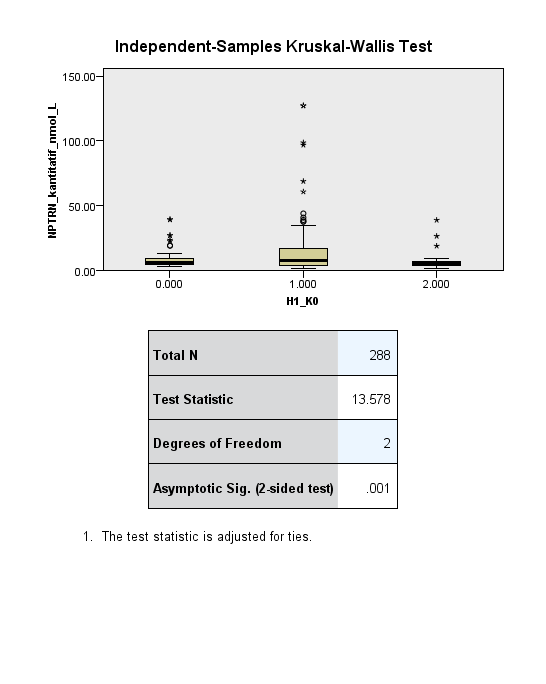


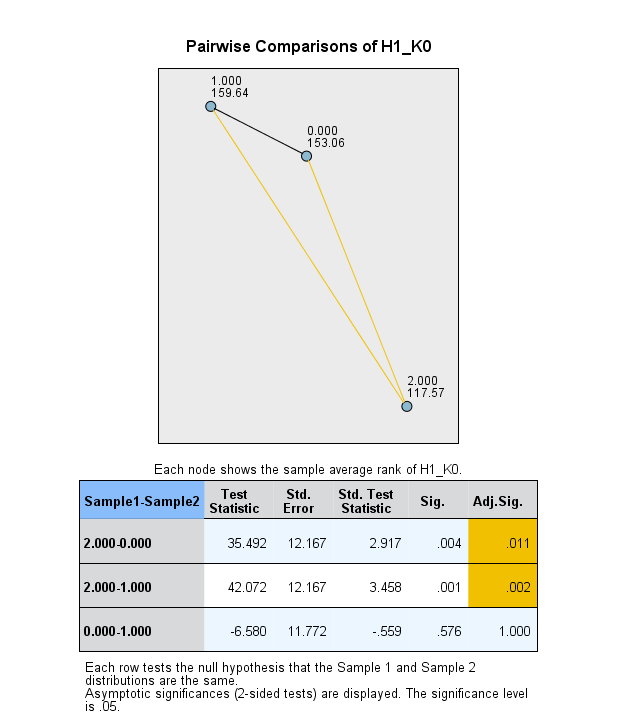


**Nonparametric Tests**

[DataSet1]


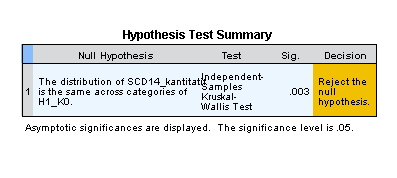


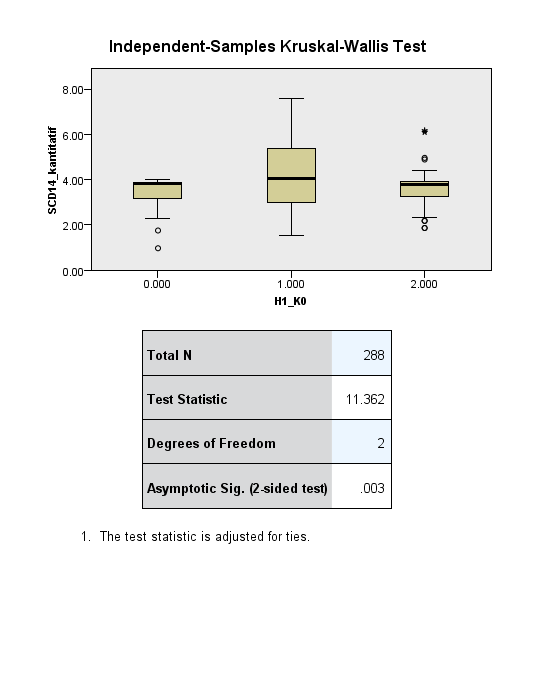


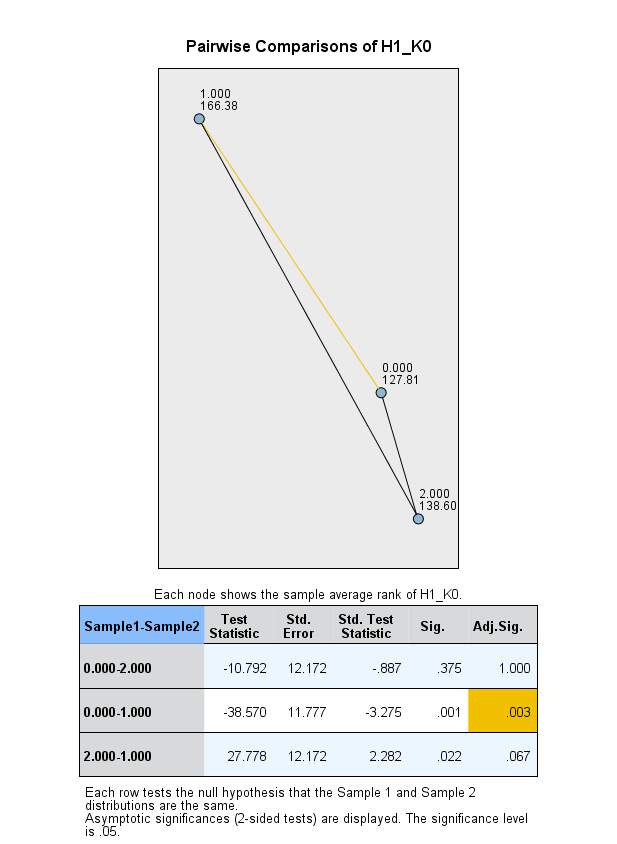


**ÖRNEKLEM BÜYÜKLÜĞÜ HESAPLAMA** (Sample size : Power Analysis)

Araştırmanın deney düzeni ve diğer koşullarına uygun olarak, NCSS paket programı kullanılarak yapılan power analizi sonucunda, ele alınan bilimsel gerçeğin saptanabilmesi için gereken denek sayısı seçenekleri önerilmiştir. Çalışma hipotezine uygun teorik kabullere uygun olduğu düşülen seçenek kullanılabilir.


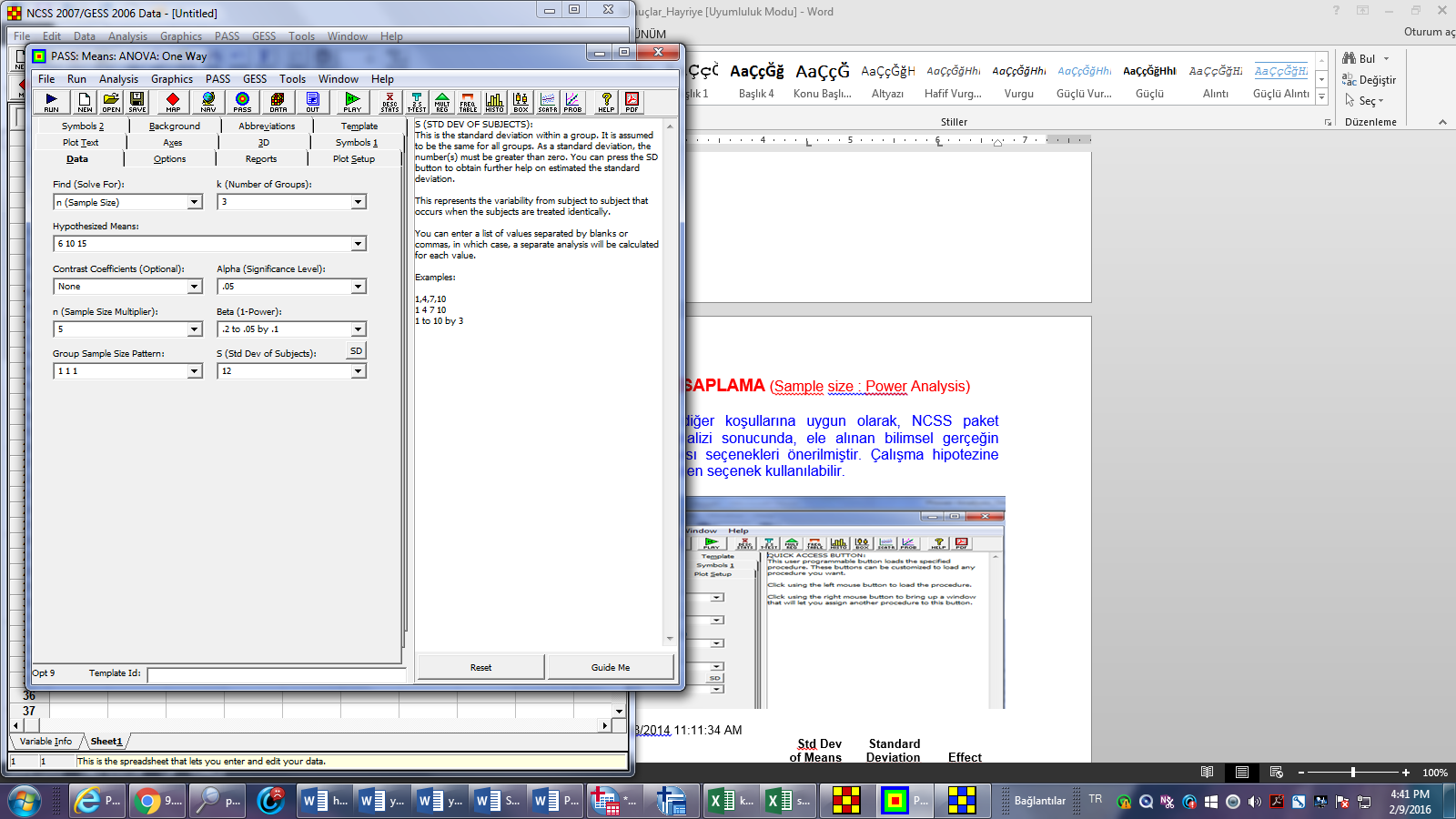


**One Way ANOVA Power Analysis**

Page/Date/Time 1 2/9/2016 4:22:04 PM

**Numeric Results**

**Std Dev Standard**

**Average Total of Means Deviation Effect**

**Power n k N Alpha Beta (Sm) (S) Size**

**0.95109 56.00 3 168 0.05000 0.04891 3.68 12.00 0.3068**

0.85292 40.00 3 120 0.05000 0.14708 3.68 12.00 0.3068

**References**

Desu, M. M. and Raghavarao, D. 1990. Sample Size Methodology. Academic Press. New York.

Fleiss, Joseph L. 1986. The Design and Analysis of Clinical Experiments. John Wiley & Sons. New York.

Kirk, Roger E. 1982. Experimental Design: Procedures for the Behavioral Sciences. Brooks/Cole. Pacific Grove,

California.

**Report Definitions**

Power is the probability of rejecting a false null hypothesis. It should be close to one.

n is the average group sample size.

k is the number of groups.

Total N is the total sample size of all groups combined.

Alpha is the probability of rejecting a true null hypothesis. It should be small.

Beta is the probability of accepting a false null hypothesis. It should be small.

Sm is the standard deviation of the group means under the alternative hypothesis.

Standard deviation is the within group standard deviation.

The Effect Size is the ratio of Sm to standard deviation.

**Summary Statements**

In a one-way ANOVA study, sample sizes of 56, 56, and 56 are obtained from the 3 groups whose

means are to be compared. The total sample of 168 subjects achieves 95% power to detect

differences among the means versus the alternative of equal means using an F test with a

0.05000 significance level. The size of the variation in the means is represented by their

standard deviation which is 3.68. The common standard deviation within a group is assumed to be

12.00.

**Details when Alpha = 0.05000, Power = 0.95109, SM = 3.68, S = 12.00**

**Percent Deviation Ni**

**Ni of From Times**

**Group Ni Total Ni Mean Mean Deviation**

1 56 33.33 6.00 4.33 242.67

2 56 33.33 10.00 0.33 18.67

3 56 33.33 15.00 4.67 261.33

ALL 168 100.00 10.33

SONUÇ; **“NPTRN_kantitatif_nmol_L”** değişkeni altgrup (Control, x1 ve x2) ortalamaları arası farkların anlamlılık denetlemesi için kullanılacak “tek yönlü varyans analizi (ANOVA)” kurgusu için yapılan power analiz sonucunda, “Tip 1 hata olasılığını (alfa anlamlılık düzeyi) 0.05, testin gücünü yaklaşık % 95 (Tip 2 hata yaklaşık 0.05 alınmıştır) ve 12 birimlik standart sapma (etki büyüklüğü yaklaşık 0.3068 alınmıştır) ön kabullerine göre her kıyas grubunda 56 denek sayısı olmak üzere toplam 168 denek gerektiği hesaplanmıştır.

Prof. Dr. Ahmet Dirican

**One Way ANOVA Power Analysis**

Page/Date/Time 2 2/9/2016 4:22:04 PM

**Chart Section**
